# AhR governs lipid metabolism: the role of gut microbiota

**DOI:** 10.3389/fmicb.2025.1442282

**Published:** 2025-01-29

**Authors:** Wanru Zheng, Mengkuan Liu, Xinyu Lv, Cuimei He, Jie Yin, Jie Ma

**Affiliations:** ^1^College of Animal Science and Technology, Guangxi University, Nanning, China; ^2^College of Animal Science and Technology, Hunan Agricultural University, Changsha, China

**Keywords:** aryl hydrocarbon receptor, gut microbiota, lipid metabolism, inflammation, metabolic disease

## Abstract

The Aryl Hydrocarbon Receptor (AhR) is widely present in mammalian bodies, showing high affinity for various exogenous substances such as polycyclic aromatic hydrocarbons (PAHs) and coumarin. Under physiological conditions, AhR mainly participates in regulating the body’s immune response, cell proliferation, and apoptosis among a series of processes. Recent studies have revealed a close connection between AhR and lipid metabolism. The gut microbiota plays a significant role in regulating host lipid metabolism. Growing evidence suggests an inseparable link between gut microbiota and AhR signaling. This review summarizes the relationship between AhR and lipid metabolism disorders, as well as the interaction between gut microbiota and AhR, exploring how this interaction modulates host lipid metabolism.

## Introduction

The aryl hydrocarbon receptor (AhR) is a ligand-dependent transcription factor that plays a crucial role in the regulatory network of the interaction between gut microbiota and the host (Hornedo-Ortega et al., 2018). Evidence suggested that AhR plays an important role in regulating metabolites involved in many biochemical pathways affecting biosynthesis and metabolism of fatty acids, bile acids, gut microbiome products, antioxidants, choline derivatives, and uremic toxins, with a central role in metabolism and signaling between multiple organs and across multiple scales (Granados et al., 2022). It was initially identified as a receptor that binds to environmental pollutant dioxins, primarily involved in detoxification and metabolic processes of dioxins and their analogs. Recent research has shown that the functions of AhR were much broader than previously understood. Further studies have shown that activating AhR influenced the differentiation, proliferation, and apoptosis of fat cells, regulating fat production (Kwack and Lee, 2000). Importantly, AhR sensed the ligands from diet, gut microbiota and host metabolites to regulate the host’s physiological functions by triggering a series of signal transduction processes. For example, Cruciferous vegetables such as broccoli, cauliflower, and cabbage can be converted into AhR ligand precursors, indole-3-carbinol (I3C) and indole-3-acetonitrile (I3ACN), through enzymatic breakdown (Ito et al., 2007). Under the action of gastric acid, these precursors further transform into high-affinity AhR ligands such as 3,3′-diindolylmethane (DIM) and indole [3,2-b] carbazole (ICZ) (Bjeldanes et al., 1991). I3C and its condensation products have potential effects in treating inflammatory bowel diseases by modulating the differentiation and function of T cells (Treg cells) through AhR activation, while reducing the number of helper T cells (Th cells) to alleviate intestinal inflammation (Rouse et al., 2013). Additionally, the plant-derived compound resveratrol can inhibit AhR activity by blocking the binding of AhR with its ligands, potentially reversing the imbalance of Th17/Treg cells in patients with autoimmune diseases and showing therapeutic potential for AhR-mediated diseases (Guo et al., 2019). *In vitro* experiments have shown that the flavonoid compound genistein can activate AhR through negative regulation of estrogen receptor alpha (ERα), promoting the expression of downstream target genes CYP1A1 and CYP1B1 (Gong F. et al., 2016; Gong P. et al., 2016). Plant extracts of the flavonoid compound cardamonin (CDN) can act as an exogenous ligand for AhR and play a crucial regulatory role in alleviating intestinal inflammation (Wang et al., 2018). Certain metabolites produced by gut microbiota such as tryptamine, indole, and their derivatives can also function as AhR ligands, inducing the production of IL-22 by intestinal immune cells and participating in gut homeostasis (Zindl et al., 2022). However, the AhR signaling mechanism by which gut microbiota regulate host lipid metabolism is unclear. Lipid metabolism disruption leads to a range of health issues such as obesity, hyperlipidemia (Li J. et al., 2016; Li M. et al., 2016), and cardiovascular diseases (Lee et al., 2005). Therefore, a thorough investigation into the mechanism by which gut microbiota modulates host lipid metabolism via AhR is beneficial for providing new insights and strategies for the prevention and treatment of related diseases.

## AhR structure

**Figure 1:**
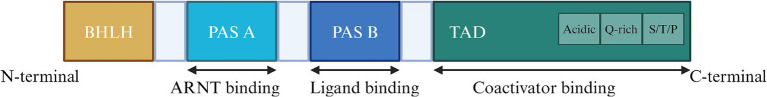
The functional domain of the AhR.

The structure of AHR determines its biological function. (Figure 1).AhR is a transcription factor whose activation relies on ligands, which is member of the basic Helix–Loop–Helix (bHLH)-Per-ARNT-Sim (PAS) family (Fukunaga et al., 1995) and its protein encoded by the AhR gene consists of 848 amino acids (Itoh et al., 1998). The structure of AhR is divided into three segments: the N-terminal, DNA-binding domain, and C-terminal (Hankinson, 1995). The AhR protein consists of three domains: bHLH, PAS, and TAD (Trans activation domain). The bHLH domain located at the N-terminus facilitates AhR binding to the promoter region of target genes and protein dimerization (Murre et al., 1989). The PAS domain is divided into PAS-A and PAS-B2, with PAS-A binding to the AhR nuclear translocator (arnt) and PAS-B binding to AhR ligands (Fukunaga et al., 1995), mediates protein dimerization. What’s more, the TAD at the C terminus is involved in protecting relevant coactivator factors. It comprises three subdomains, with the first two subdomains are enriched in acidic residues and glutamine, while the third subdomain is enriched in serine, threonine, and proline (S/T/P) (Lin et al., 2022).

The BHLH domain is located at the N terminus, initiates AhR binding and mediating protein dimerization, the PAS domain is the binding site for ARNT and AhR ligands, and the TAD at the C terminus, involved in transcription activation, containing three subdomains, the first rich in acidic residues, the second rich in glutamine, and the third rich in serine, threonine, and proline.

## AhR expression

AhR exists in the form of a cytoplasmic protein complex composed of HSP90, p32, and XAP-2 within the cytoplasm of cells (Zhu et al., 2021), translocates to the nucleus upon activation by agonists and binds to aryl hydrocarbon receptor nuclear translocator (ARNT) or hypoxia-inducible factor 1β (HIF-1β), which interacts with xenobiotic response elements (XREs) to control the expression of key genes (Bahman et al., 2024). AhR is present in various tissues and cells of vertebrates, such as the intestines, liver, spleen, lymph nodes, and is expressed in various types of cells in the body, including immune cells, epithelial cells, endothelial cells, and stromal cells (Stockinger et al., 2014). Among these, immune cells are one of the main sites of AhR gene expression, especially macrophages, dendritic cells, and T lymphocytes (Trikha and Lee, 2020), which play an important role in immune responses and inflammatory reactions. Li et al. discovered that the AhR signal plays a significant regulatory role in the expression of CD117 on the surface of ILC3 (type 3 innate lymphoid cells), and in patients with Crohn’s disease (CD), attenuation of the AhR signal can lead to the transformation of ILC3 into ILC1, thereby increasing inflammation in the terminal ileum (Li J. et al., 2016; Li M. et al., 2016). Climaco-Arvizu et al. reported that AhR could regulate the differentiation of IBD intestinal macrophages. Loss of the AhR gene enhances inflammatory M1 polarization of macrophages, weakens anti-inflammatory M2 polarization, and affects the production and secretion of inflammatory factors, thereby regulating inflammation development (Climaco-Arvizu et al., 2016). In addition to immune cells, epithelial cells and endothelial cells are also important sites of AhR gene expression (Major et al., 2023; Juan et al., 2006). Then researchers found that endothelial cells have higher levels of AhR expression compared to immune cells and epithelial cells through immunofluorescence detection techniques (Major et al., 2023). Further studies showed that AhR was expressed in many types of lung cells, and the cells with high expression mainly included lung endothelial cells and alveolar cells, affecting lung barrier function (Pang et al., 2017).

## Gut microbiota regulates lipid metabolism by AhR signal

**Figure 2:**
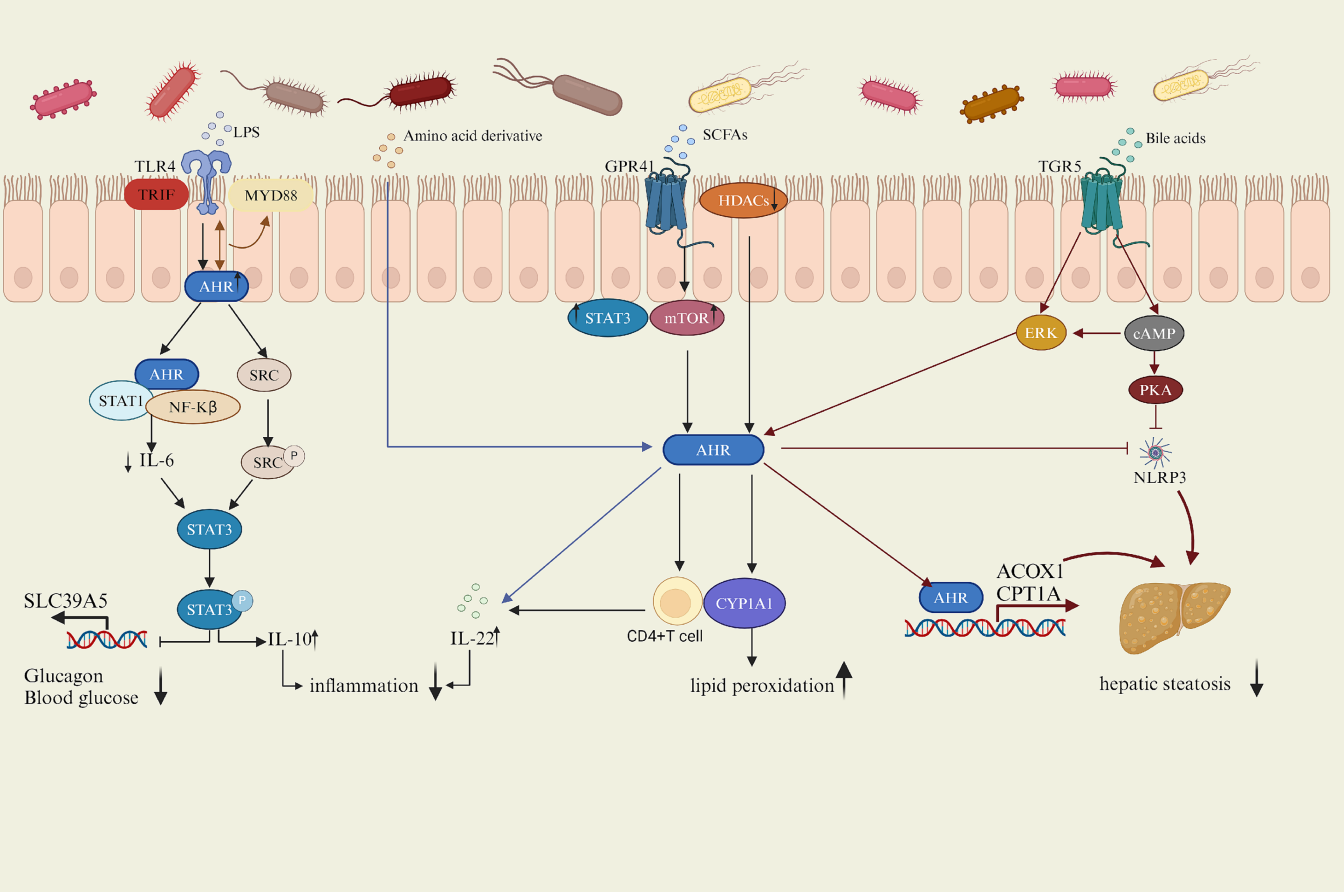
Mechanism of AhR regulation of lipid metabolism.

Increasing evidence suggested that AhR played a crucial role in regulating lipid metabolism, with the gut microbiota influencing AhR activity through its metabolites such as lipopolysaccharide (LPS), amino acid derivative, short-chain fatty acids (SCFAS) and bile acids (BAS) or direct interactions, thereby modulating the host’s physiological processes (Figure 2). This article will explore the potential mechanisms by which the gut microbiota affects host lipid metabolism via the AhR signaling pathway, focusing on the gut microbiota itself and its metabolites influencing AhR activity.

AhR activation reduces IL-6 secretion by inhibiting the NF-κB pathway induced by LPS, IL-6 activates stat3 and inhibits the transcription and expression of zinc transporter SLC39A5, AhR enhances IL-10 levels by upregulating Src-STAT3 signaling triggered by LPS, alleviating lipid metabolism disorders. SCFAs exacerbates lipid peroxidation by inhibiting HDACS and increasing the expression of CYP1A1. SCFAs increase the AhR expression in CD4 T cells by activating STAT3 and mTOR signaling pathways after binding to the GPR41 and then promoted the production of IL-22; amino acids also promote IL-22 production after being metabolized by microorganisms into AhR ligands, and the increase of IL-22 improves hyperglycemia. BAs bind to TGR5 to increase AHR expression and promote transcription of ACOX 1 and CPT 1 A by triggering CAMP-ERK pathway and inhibit NLRP3 activity by triggering CAMP-PKA pathway, while AHR also inhibit activation of NLRP3, thus inhibiting hepatic steatosis.

### LPS and AhR

LPS is a complex sugar-lipid-protein compound and a major component of bacterial endotoxins found widely in the outer walls of Gram-negative bacteria. Inside the host, LPS is recognized by the immune system as a pathogen-associated molecular pattern (PAMP), triggering an inflammatory response, and classical signaling involves the Toll-like receptor 4 (TLR4)(Facchini et al., 2020), which is a protein associated with the immune system and inflammatory responses, mainly expressed in lymphocytes, macrophages, endothelial cells, and cardiomyocytes (Biemmi et al., 2020), playing a crucial role in the host’s immune system. Activation of TLR4 triggers MyD88-and TRIF-dependent signaling pathways (Sun et al., 2019). It recruits myeloid differentiation factor 88 (MyD88) and activates MyD88-dependent NF-κB signaling pathway, inducing the production of inflammatory factors (Wang B. et al., 2023; Wang Y. et al., 2023). AhR was expressed in peritoneal macrophages stimulated by LPS, being induced by TLR signaling. In the LPS signaling pathway, AhR can negatively regulate it by interacting with Stat1. In macrophages, the aryl hydrocarbon receptor (AhR) is activated, forming a complex with the transcription factors STAT1 and NF-kB, inhibiting NF-kB-mediated downstream factor IL-6 transcription, resulting in suppressed IL-6 expression, thus alleviating LPS-induced inflammatory response (Kimura et al., 2009). IL-6, as a pro-inflammatory factor, whose down-regulation inhibited hepatocyte adipogenesis and reduced macrophage inflammatory response. Blocking IL-6 signaling reduced the occurrence of NAFLD (Park et al., 2023) and inhibited obesity-related ventricular arrhythmias (Aromolaran et al., 2024). Moreover, IL-6 activated stat3, increased stat3 phosphorylation, and inhibited the transcription and expression of zinc transporter SLC39A5, thereby increasing glucagon secretion and the risk of T2D (Chen et al., 2023). Furthermore, LPS also triggered the AhR-mediated activation of the Src-STAT3 signaling pathway (Zhu et al., 2018). AhR in the cytoplasm upregulates the tyrosine phosphorylation of Src kinase (Src). Src, as a non-receptor tyrosine kinase, participated in various cellular signaling transduction processes, significantly affecting cell growth, proliferation, and differentiation (Brown and Cooper, 1996), catalyzing the phosphorylation of STAT3 (signal transducer and activator of transcription3) and leading to the activation of STAT3. Then STAT3 translocated to the nucleus and regulated the transcriptional expression of relevant genes. Src-STAT3 signaling pathway further promotes the secretion of IL-10 through AhR mediation, collectively inhibiting the inflammatory phenotype of macrophages. IL-10, an anti-inflammatory cytokine, suppresses the production and release of various inflammatory mediators, thereby attenuating the metabolic inflammation (Zhu et al., 2018). Adipose tissue-derived stem cells (ADSCs) promoted the expression of IL-10 to ameliorate hyperglycemia and insulin resistance and prevented T2D (Zhang et al., 2017). Overexpression of IL-10 had also been shown to restore intestinal repair after HFD feeding, normalizing barrier repair in HFD-treated mice (Hill et al., 2023). These studies indicated that the expression level of IL-10 is closely related to lipid metabolism. Interestingly, AhR was also shown to have an interaction with TLR4 and together regulate the downstream factor MyD88 (Zhang et al., 2023). These results indicate an inhibitory effect on the LPS-induced inflammatory response by enhancing the activation of AhR, suggesting that AhR agonists such as related ligands or probiotics can be used in clinical application to mitigate the inflammatory effects of LPS. For example, the AhR endogenous ligand indole-3-lactic acid (ILA) significantly attenuated NF-κB activation in macrophages (Calzetta et al., 2022) and *Bacillus amyloliquefaciens* alleviated LPS-induced intestinal inflammation through the AhR/STAT3 pathway (Wang et al., 2024).

### SCFAs and AhR

Short-chain fatty acids (SCFAS) are metabolites generated by intestinal flora through biotransformation, mainly including acetic acid, propionic acid and butyric acid (He et al., 2020), in which Bacteroides mainly produce acetic acid and propionic acid, while Firmicutes mainly produce butyric acid (Macfarlane and Macfarlane, 2003). After SCFAS are uptaken by the intestine, it undergoes a series of transformation processes in the liver, mainly producing acetyl-CoA and propionyl-CoA, which participate in several biological metabolic pathways, such as glycogen synthesis, gluconeogenesis process and cholesterol synthesis, and then affect the host lipid metabolism (den Besten et al., 2013). SCFAs are reported to enhance gene expression mediated by the aromatic hydrocarbon receptor (AhR) and significantly increase the expression of AhR response genes such as Cytochrome P450 1A1 (CYP1A1) by inhibiting the activity of histone deacetylases (HDACs) (Jin et al., 2017). Interestingly, overexpression of CYP1A1 exacerbated lipid peroxidation in the NAFLD model (Huang et al., 2018). Moreover, butyrate also increased the AhR expression in CD4 T cells by activating the STAT3 and the mammalian target of rapamycin (mTOR) signaling pathways after binding to the G-protein-coupled receptor 41 (GPR41) and then promoted the production of IL-22 (Yang et al., 2020). IL-22 plays an important role in lipid metabolism, as it improves insulin sensitivity, protects the intestinal mucosal barrier, reduces endotoxemia and chronic inflammation, IL-22 receptor-deficient and high-fat-fed mice are prone to metabolic disorders, while administration of exogenous IL-22 to obese mice reversed the induced symptoms like hyperglycemia and insulin resistance (Wang et al., 2014).

### BAs and AhR

Bile acids are vital signaling molecules synthesized from cholesterol in the liver, with the classic pathway of their synthesis triggered by cholesterol 7α-hydroxylase (CYP7A1) catalyzing cholesterol 7α-site hydroxylation (Ahmad and Haeusler, 2019). The host often promotes the utilization of bile acids through enterohepatic circulation, approximately 95% of primary bile acids are reabsorbed at the terminal ileum and return to the liver via the portal vein; a small portion of primary bile acids are catalyzed by BAs salt hydrolase (BSH) enzymes from gut microbiota into free BAs, which undergo conversion into secondary bile acids through pathways like dehydrogenation and dehydroxylation under the influence of intestinal flora (Hsu and Schnabl, 2023). Microorganisms expressing BSH are primarily members of the Firmicutes phylum (Jones et al., 2008), disturb the gut ecology, while in IBD, BSH-producing Firmicutes were reduced (Ramos and Papadakis, 2019), impeding the conversion of PBAs to SBAs and thereby affecting bile acid metabolism. Intraepithelial lymphocytes (IELs) play a protective role in IBD models (Panda et al., 2023), Furthermore, AhR regulated the development, proliferation, and function of intraepithelial lymphocytes (IELs) (Li et al., 2011), which may be beneficial for gut microbial balance and Maintenance of firmicutes diversity in IBD, thus enhancing secondary bile acid generation. SBAs inhibited the expression of pro-inflammatory genes by activating the membrane receptor TGR5 (Duboc et al., 2013), suggesting a potential synergistic effect between bile acids and AhR in suppressing inflammatory responses. TGR5 activation on ciliated and non-ciliated bile ducts triggered downstream signaling pathways such as expression of cAMP, AKT, and extracellular signal-regulated kinase (ERK) (Guo et al., 2016). Meanwhile TGR5 as a membrane-bound receptor played a significant role in glucose metabolism (Hui et al., 2020), lipid metabolism (Arab et al., 2017), and anti-inflammatory immune regulation (Chiang, 2013). For example, TGR5 suppressed the activation of the NLRP3 inflammasome by activating intracellular signaling pathways, particularly the cAMP-PKA axis (Tian et al., 1999). NLRP3 inflammasome, a crucial innate immune molecule, promotes the release of pro-inflammatory factors and exacerbates inflammatory responses when activated. Studies on TGR5−/− mouse models show that genetic deficiency leads to overactive NLRP3 inflammasomes, resulting in elevated pro-inflammatory factors and enhanced M1 polarization of macrophages in adipose tissue (Shi et al., 2021), exacerbating inflammation. While the inhibition of NLRP3 inflammasome activity reduced liver inflammation and fibrosis and improved NAFLD (Shen Q. et al., 2023; Shen X. et al., 2023). As a negative regulator of NLRP3 inflammasomes, AhR inhibited the activation of NLRP3 inflammasomes, the reason is that AhR bound to its endogenous ligand and inhibited NF-κB transcription, leading to reduced NLRP3 transcription (Huai et al., 2014; Qiao et al., 2022). Furthermore, TGR5 activated the ERK to induce phosphorylation of dynamin-related protein 1 (Drp1) in mitochondria dynamics. Activation of the ERK pathway can induce phosphorylation of Drp1 (Prieto et al., 2016), leading to mitochondrial fission, increasing the rate of fatty acid beta-oxidation while reducing fat accumulation. Additionally, cAMP is also involved in ERK signal transduction (Enserink et al., 2002). In cilia-related liver disorders such as autosomal dominant and autosomal recessive polycystic kidney diseases, cAMP levels are elevated and TGR5 is overexpressed in cholangiocytes but not localized on cilia. In ciliary cholangiocytes, TGR 5 agonists reduced cAMP levels and cell proliferation, but ERK signaling was activated, and the cAMP levels also affected the phosphorylation of ERK (Masyuk et al., 2013). The activation of ERK promoted AHR expression, then AHR directly binded to the promoter regions of the key fatty acid oxidation enzymes ACOX 1 and CPT1A to transcribe and activate their expression and then achieved normal fatty acid oxidation function, thus inhibiting hepatic steatosis (Han et al., 2021). Interestingly the upregulation of ERK signaling can inhibit AhR expression (Jiang et al., 2021), suggesting that activation of ERK pathway can inhibit AhR signaling and thus affect lipid metabolism.

### Amino acids (AAs) and AhR

Most amino acids are absorbed in the small intestine, while those not absorbed enter the colon to participate in microbial metabolism processes, leading to the production of various metabolites such as ammonia, amines, short-chain fatty acids, branched-chain fatty acids, hydrogen sulfide, organic acids, and phenols (Abdallah et al., 2020; Ma and Ma, 2019). Studies indicate that branched-chain amino acids and aromatic amino acids play crucial roles in lipid metabolism disorders associated with obesity, insulin resistance, diabetes, and fatty liver (Ejtahed et al., 2020). And aromatic amino acids mainly include phenylalanine, tyrosine, and tryptophan, which activate the AhR to induce downstream pathway alterations (Yang et al., 2019). The ability of gut microbes to metabolize tryptophan is reduced, which lowering the activation of AhR to promote metabolic disease (Natividad et al., 2018), suggesting that AhR may regulate lipid metabolism through the gut microbiota. Tryptophan is a crucial source of endogenous AhR ligand precursors (Liu et al., 2021) and its metabolites such as kynurenine and the photoproduct 6-formylindolo [3,2-b] carbazole (FICZ), can bind to the aryl hydrocarbon receptor (AhR) in the intestine, thereby regulating the function and differentiation of intestinal immune cells (Gutiérrez-Vázquez and Quintana, 2018). Kynurenine promoted the differentiation of CD4+ naïve T cells into anti-inflammatory Treg cells (Mezrich et al., 2010), while FICZ, as a high-affinity ligand for AhR, activated the AhR signaling pathway at extremely low concentrations (Rannug and Fritsche, 2006), upregulating the expression of the cytochrome P450 family 1.

(CYP1) family of genes. Further CYP1A1 rapidly degraded FICZ, forming a negative feedback regulatory mechanism to ensure a low level of FICZ in the gastrointestinal tract (Wei et al., 2000). However, FICZ also induced the differentiation of Th17 cells and the expression of the inflammatory factor IL-17, which inhibited the differentiation of Treg cells (Quintana et al., 2008). Studies have shown that gut microbiota metabolized tryptophan into indole and its derivatives, thereby participating in the regulation of AhR signaling. For instance, tryptophan was metabolized by *Lactobacillus reuteri* to indole-3-aldehyde (Zelante et al., 2013). In mouse models, this substance can activate AhR and induce the production of IL-22, which plays a crucial role in maintaining intestinal mucosal immune homeostasis. Additionally, tryptophan metabolites from other commensal microbiota, such as indole-3-acetic acid, indole-3-aldehyde, tryptamine, and 3-methylindole (Shen et al., 2022; Dang et al., 2023), also exhibit AhR agonist activity, suggesting a potentially significant role in intestinal immune regulation. The microbial community in the gut generates AhR agonists during tryptophan metabolism, supporting the growth and development of ILC3 in the intestine. AhR is an essential transcription factor for ILC3 (Li J. et al., 2016; Li M. et al., 2016), ILC3 is a critical member of the intestinal mucosal immune system, and the dysfunction of ILC3s may lead to inflammatory diseases in intestinal mucosal tissues (Cording et al., 2018). What’s more, ILC3 protected the body from damage by the symbiotic microbiome through producing key anti-inflammatory factors, such as IL-22 and IL-17A (Shen Q. et al., 2023; Shen X. et al., 2023), to prevent inflammation in adipose tissue.

## AhR and lipid metabolism disorder

AhR plays a crucial role in numerous biological processes, including immune responses, cell proliferation and differentiation, as well as maintaining homeostasis (Wang et al., 2022). However, an increasing number of studies indicates that AhR plays an important role in lipid metabolism, causing lipid metabolic diseases such as obesity (Kerley-Hamilton et al., 2012), non-alcoholic fatty liver disease (NAFLD) (Moyer et al., 2017), and type 2 diabetes (T2D) (Wang et al., 2011). The expression of lipid metabolism-related phenotypes in these disease models can be promoted or inhibited by adjusting AhR levels. (Table 1) Therefore, it is necessary to study the effect of AhR on lipid metabolism in detail in order to use it as a potential therapeutic target for lipid metabolism diseases.

**Table 1:** Effect of AhR expression level on phenotype related to lipid metabolic diseases.

Disease	Model	AhR level	phenotypes	Reference
Obesity	Mouse	Inhibit	WAT ↓ Cyp1a1 ↓ PPARƴ ↓ Foxo1 ↓ Scd1 ↓ Spp1 ↓ CYP4A ↓ FGF21 ↓ FADS1 ↓ ELOVL5 ↓ IL-6 ↓ STAT3 ↓	Moyer et al. (2017), Xu et al. (2015), Huang et al. (2022)
	Human	Elevate	Cyp1b1 ↑ Leptin ↑ TC ↑ TG ↑ LDL-C ↑ Ghrelin ↓ Scd1 ↓ PPARƴ2 ↓ ACC2 ↓ CPT1a ↓	Shahin et al. (2020)
NAFLD	Mouse	Inhibit	ALT ↓ AST ↓ TG ↓ TC ↓ Cyp1a1 ↓ TNF-a ↓	Xia et al. (2019)
	HepG2	Inhibit	MDA ↓ ROS ↓ SOD ↑	Xia et al. (2019)
T2D	Mouse	Inhibit	PPARα ↓ Aco ↓ Cpt1b ↓ Pepck ↓ G6pase ↓ Hepaticglycon↑ pAKT(Ser473)↑ Cyp1a1 ↓ Cyp1b1 ↓	Wang et al. (2011), Xia et al. (2019), Jaeger et al. (2017)
	Mouse	Elevate	pAKT ↓ pNFκB ↑ ICAM ↓ INOS ↓ FMO3 ↓ IL-10 ↑ IL-22 ↑ TG ↓ ALT ↑ GLP-1 ↑	Liu et al. (2020), Lin et al. (2019), Natividad et al. (2018)

### AhR and obesity

With the improvement of living standards, obesity is becoming more common and prevalent worldwide. Obesity refers to the excessive accumulation of fat in the body, mainly caused by the excessive accumulation of triglycerides in the body (Twig et al., 2020). Typically characterized by exceeding the normal weight range and an increase in body fat percentage, obesity is associated with various chronic diseases and health issues, severely affecting the quality of life (Piché et al., 2020). Therefore, finding effective methods to address obesity is crucial. Obesity affected the diversity of the gut microbiota, with a decrease in the abundance of Bacteroidetes and increased proportion of Firmicutes in obese individuals, suggesting a possible role in regulating obesity by remodeling gut microbial community structure (Ley et al., 2005). Studies have shown that the activation of AhR induced obesity (Kerley-Hamilton et al., 2012), It may be because the aryl hydrocarbon receptor repressor (AhRR) was significantly down-regulated in obese populations, while AhR and CYP1B1 are significantly upregulated, indicating that AhRR may regulate obesity by inhibiting AhR expression through the AhR-CYP1B1 axis (Shahin et al., 2020). AhR deficiency significantly reduced weight gain and adiposity, increasing the protein and mRNA expression of fibroblast growth factor 21 (FGF21), which activates thermogenesis in brown adipose tissue (BAT) and gWAT, thus increasing metabolic rate and energy expenditure, preventing obesity induced by a high-fat diet (Girer et al., 2019), making it a potential target for obesity treatment. Studies have shown that Kynurenine caused obesity by activating AHR (Moyer et al., 2016), while obesity and hepatic steatosis were prevented by inhibiting AhR, the AhR antagonist naphthoflavone (aNF) prevented and reversed obesity in high-fat diet mice by inhibiting AhR and related genes in its network, such as CYP1B1 and stearoyl-CoA desaturase-1 (SCD1) (Moyer et al., 2017; Xu et al., 2015). CYP1B1 is a member of the cytochrome P450 superfamily that is involved in metabolizing endogenous compounds including steroid hormones and lipids, which regulate metabolism, accumulation, and distribution in adipose tissue. The expression of CYP1B1 influenced the development of obesity, in CYP1B1-null mice, the expression level of SCD1 was reduced, which inhibited obesity and thus affected lipid metabolism (Li et al., 2014). Further research found that SCD1 is a delta-9 fatty acid desaturase that catalyzes the synthesis of monounsaturated fatty acids. Similarly, SCD1-deficient mice reduced lipid synthesis and enhanced insulin sensitivity, promoting the suppression of obesity (Flowers and Ntambi, 2008). In obese patients, tryptophan was preferentially catabolized through the kynurenine pathway (KP), leading to an excessive increase in the concentration of kynurenine (Kyn) in the blood, which activated AhR and then transcribed STAT3 expression to enhance the secretion of IL-6 (Huang et al., 2022), which maintained the proliferation rates of obese adipose tissue (Ackermann et al., 2024). In contrast, knockdown of AhR from adipocytes abolished the effects of Kyn and prevented obesity.

### AhR and NAFLD

NAFLD refers to the pathological condition where the liver accumulates fat without excessive alcohol consumption. NAFLD is the most common chronic liver disease (Zhou et al., 2020) and a significant component of metabolic syndrome (Haas et al., 2016), which included obesity, insulin resistance, hypertension, hypertriglyceridemia, and low high-density lipoprotein cholesterolemia (Chen et al., 2012). The spectrum of NAFLD ranges from simple steatosis (fatty liver) to non-alcoholic steatohepatitis (NASH), fibrosis, and cirrhosis (Schuster et al., 2018). Activation of the AhR has been shown to affect lipid metabolism in the liver, including synthesis and oxidation of fatty acids. Abnormal fat accumulation in the liver is a primary feature, and AhR activation can induce lipid deposition, potentially directly impacting the pathogenesis of NAFLD (Podechard et al., 2009). It has been observed that the activation of AhR signaling pathway indirectly induced the accumulation of lipid droplets in rat hepatocytes (Neuschäfer-Rube et al., 2015). The AhR-CYP1A1 signaling pathway was activated to cause intracellular lipid droplet accumulation in Hepatitis C virus (HCV) (Ohashi et al., 2018). As an exogenous ligand for the AhR, the Sulforaphane (SFN) can regulated the intestinal microflora of high-fat diet mice to prevent NAFLD by activating the AhR/SREBP-1C pathway, reduced the protein levels of indole-3-acetic acid (IAA), sterol regulatory element-binding protein-1c (SREBP-1C), acetyl-CoA carboxylase 1 (ACC1), and fatty acid synthase (FAS), And then regulates hepatic lipid metabolism, And to prevent NAFLD (Xu et al., 2021). Further studies showed that AhR promoted the absorption of fatty acids by activating its transcriptional target CD36.(Lee et al., 2010). Overexpression of the AhR in the liver significantly upregulates the expression of the fatty acid translocase (FAT) CD36 in mouse liver cells, promoting the uptake of fatty acids by liver cells (Yao et al., 2016), which exacerbated lipid deposition in the liver, leading to liver damage and promoting the development of NAFLD. AhR ligand 3-methylcholanthrene (3MC) also significantly increased the expression level of fatty acid translocase in liver by activating AhR, inducing hepatic steatosis (Kawano et al., 2010). Estrogen deficiency is one of the main causes of obesity and NAFLD (Zhu et al., 2020). However, the administration of endogenous agonists of AhR such as cinnabarinic acid (CA) down-regulated CD36 and reduced the uptake of free fatty acids in hepatocytes, thus achieving the inhibition of hepatic steatosis and liver injury (Patil et al., 2022). Importantly, CYP1A1 is an estrogen-metabolizing enzyme, and increased activity of CYP1A1 leads to estrogen deficiency (Niwa et al., 2015), for example, Benzo[a]pyrene (Bap) promoted the transcription and overexpression of CYP1A1 by activating the AhR pathway, inhibiting estrogen’s protective effect on the liver, significantly increasing the risk of NAFLD (Mumtaz et al., 2022). In addition, alpha-naphthoflavone, as an AhR inhibitor, alleviated NAFLD by inhibiting the AhR-CYP1A1 pathway (Xia et al., 2019). The AhR-CYP1A1 axis regulates lipid peroxidation by influencing the level of reactive oxygen species (ROS) and superoxide dismutase (SOD) (Huang et al., 2018). When the expression of AhR increases, the CYP1A1 also increases to enhance ROS (Cui et al., 2024) while excess ROS will lead to the production of lipid peroxides such as malondialdehyde (MDA), which may exacerbate oxidative stress and mitochondrial damage (Wang B. et al., 2023; Wang Y. et al., 2023) and promote the production of NAFLD (Zhao et al., 2023).

### AhR and T2D

T2D is the most common type of diabetes globally, accounting for over 90% of all diabetes cases. Unlike type 1 diabetes, T2D is characterized by insulin resistance and/or dysfunction of pancreatic *β*-cells, leading to sustained high blood sugar levels and the former is due to autoimmune destruction of insulin-producing cells (ElSayed et al., 2023). Abnormal expression of AhR will result in imbalanced glucose and lipid metabolism, indicating a crucial role of AhR in regulating these processes in the body (Biljes et al., 2015). Thus, AhR may be a key factor in the development of diabetes. The development of T2D is associated with a state of chronic low-grade inflammation (Gong F. et al., 2016; Gong P. et al., 2016). In high sugar-induced insulin resistance and diabetes complications, AhR is crucial for maintaining ILC3, promoting the development and maturation of ILC3, and stimulating the secretion of IL-22 by ILC3 to inhibit inflammation levels, thereby preserving intestinal homeostasis (Artis and Spits, 2015; Kobayashi et al., 2014). IL-22 released by ILC3 cells protected pancreatic islet beta cells from inflammation and glucotoxicity, potentially reversing the damage caused by hyperglycemia to pancreatic islet beta cells, thus improving insulin sensitivity (Abadpour et al., 2018). Furthermore, AhR ligands enhanced intestinal defense mechanisms, reduced bacterial translocation and systemic inflammation, effectively reversing glucose intolerance and insulin resistance induced by diabetes (Liu et al., 2020). The indirubin, an AhR agonist, induced the secretion of IL-10 and IL-22 by activating AhR to prevent high-fat diet-induced insulin resistance in mice model (Lin et al., 2019). Other AhR agonists, such as indoles, have been shown to effectively stimulate the secretion of glucagon-like peptide-1 (GLP-1), thus improving insulin resistance and alleviate symptoms of T2D (Natividad et al., 2018). Further studies have found that tryptophan, as a ligand of AHR, was metabolized by gut microbiota into 5-hydroxyindole-3-acetic acid (5-HIAA) promoting the ubiquitin–proteasome degradation of Suv39h1 by activating AhR, thereby stimulating TSC2 expression and inhibiting the activation of mTORC1 signaling, which would promote insulin signaling, improve glucose intolerance and reduce the risk of T2D (Du et al., 2024). However, it has also been shown that lack of AhR improved insulin sensitivity and glucose tolerance (Wang et al., 2011) by increasing energy expenditure and ameliorating high-fat diet-induced insulin resistance in mice (Jaeger et al., 2017). Although these results are inconsistent, the important role of AhR cannot be ignored. Thus, further investigation and confirmation are needed on how AhR specifically affects glucose metabolism and T2D.

### Limitations and future direction

Increasing evidences demonstrate that AhR signaling is associated with lipid metabolism, and although some significant progress has been gained about AhR regulating lipid metabolism, translating these findings into clinical treatment and preventive strategies still faces many challenges. The gut microbiome is highly diverse, containing thousands of different microbial species. There are complex interrelationships among these microorganisms, including symbiosis, competition and antagonism. Even though certain microorganisms have been found to be associated with AHR activation and lipid metabolism changes, the role of these microorganisms may be altered by the influence of other microorganisms in the context of the entire microbial community. AHR is a pleiotropic transcription factor, in addition to regulating the genes involved in lipid metabolism. It is also involved in many other biological processes, such as immune response, cell proliferation, and differentiation. AHR can regulate the expression of numerous genes that may have different functions in different cell types and physiological conditions. Therefore, it is difficult to distinguish between the direct and indirect effects of AHR on lipid metabolism and how these effects are synergized in complex physiological and pathological processes.

At present, relevant studies mainly focus on animal models and cell experiments, such as mice, rats and liver cell lines (Zhao et al., 2022). While these studies provide us with valuable experimental evidence, there are certain physiological and metabolic differences between animal models and humans, so these results may not fully reflect the real situation in the human body. In addition, different research teams use different experimental conditions and methods, resulting in a certain diversity of research results, which makes it difficult to interpret and apply these results. Some studies have shown that AhR is protective against diet-induced metabolic syndrome (Wada et al., 2016), while others are negative (Korecka et al., 2016). The AhR signaling pathway involves multiple molecular and cellular processes, so the experimental design needs to be highly precise and complex to accurately simulate what is really happening *in vivo*. However, these complex interactions may not be fully captured by the current experimental methods, the specific molecular mechanisms and signaling pathways still require further intensive investigation.
